# Association between Amylin and Amyloid-β Peptides in Plasma in the Context of Apolipoprotein E4 Allele

**DOI:** 10.1371/journal.pone.0088063

**Published:** 2014-02-10

**Authors:** Wei Qiao Qiu, Max Wallack, Michael Dean, Elizabeth Liebson, Mkaya Mwamburi, Haihao Zhu

**Affiliations:** 1 Departments of Psychiatry, Boston University School of Medicine, Boston, Massachusetts, United States of America; 2 Pharmacology and Experimental Therapeutics, Boston University School of Medicine, Boston, Massachusetts, United States of America; 3 Alzheimer's Disease Center, Boston University School of Medicine, Boston, Massachusetts, United States of America; 4 McLean Hospital, Harvard Medical School, Belmont, Massachusetts, United States of America; 5 Department of Public Health and Family Medicine, Tufts University, Boston, Massachusetts, United States of America; Cleveland Clnic Foundation, United States of America

## Abstract

Amylin, a pancreatic peptide that readily crosses the blood brain barrier (BBB), and amyloid-beta peptide (Aβ), the main component of amyloid plaques and a major component of Alzheimer's disease (AD) pathology in the brain, share several features. These include having similar β-sheet secondary structures, binding to the same receptor, and being degraded by the same protease. Thus, amylin may be associated with Aβ, but the nature of their relationship remains unclear. In this study, we used human samples to study the relationship between plasma amylin and Aβ in the context of the apolipoprotein E alleles (ApoE). We found that concentrations of Aβ1-42 (P<0.0001) and Aβ1-40 (P<0.0001) increased with each quartile increase of amylin. Using multivariate regression analysis, the study sample showed that plasma amylin was associated with Aβ1-42 (β = +0.149, SE = 0.025, P<0.0001) and Aβ1-40 (β = +0.034, SE = 0.016, P = 0.04) as an outcome after adjusting for age, gender, ethnicity, ApoE4, BMI, diabetes, stroke, kidney function and lipid profile. This positive association between amylin and Aβ1-42 in plasma was found regardless of the ApoE genotype. In contrast, the relationship between amylin and Aβ1-40 in plasma seen in ApoE4 non-carriers disappeared in the presence of ApoE4. Using AD mouse models, our recent study demonstrates that intraperitoneal (i.p.) injection of synthetic amylin enhances the removal of Aβ from the brain into blood, thus resulting in increased blood levels of both amylin and Aβ. The positive association between amylin and Aβ, especially Aβ1-42, in human blood samples is probably relevant to the findings in the AD mouse models. The presence of ApoE4 may attenuate amylin's capacity to remove Aβ, especially Aβ1-40, from the AD brain.

## Introduction

Amylin is a short peptide of 37 amino acids produced and secreted by the pancreas. Amylin and amyloid-beta peptide (Aβ), the main component of amyloid plaques and a major component of brain Alzheimer's disease (AD) pathology, share several features, including similar β-sheet secondary structures [1], binding to the same amylin receptor [2], and being degraded by the same protease insulin-degrading enzyme (IDE) [3]–[5]. They appear to affect each other in complex ways. A recent study found an accumulation of amylin amyloid in the cerebrovascular system in the AD brain, resulting in impaired vascular functioning [6]. Amylin readily penetrates the blood brain barrier (BBB) [7], [8] and mediates important brain functions including inhibiting appetite thereby improving glucose metabolism[9], [10], relaxing cerebrovascular structure [11], [12], and, in all likelihood, enhancing neural regeneration [13]. High levels of Aβ in the AD brain may block amylin's ability to bind to its receptor, thus hindering normal amylin functions that are essential to the brain [10].

Our recent study using two AD mouse models demonstrates another important function of amylin in the brain. Chronic treatment with intraperitoneal (i.p.) injection of amylin or its clinical analog, pramlintide, enhanced removal of Aβ from the brain and improved their cognitive impairment (submitted and under review). Through efflux, Aβ can pass through the BBB into blood [14]. BBB dysfunction, decreased cerebral blood flow, and impaired vascular clearance of Aβ from the brain are all thought to contribute to AD pathogenesis [15]. It is well known that the concentration of Aβ in blood is much lower than the concentration of Aβ in the brain [16], suggesting that only a small portion of Aβ in the brain can be removed from the brain. As Aβ is a key element of AD pathogenesis in the brain [17], if a drug or substance like amylin or its analogs can enhance the removal of Aβ from the AD brain into the blood, it might prove an effective treatment for the disease. The use of solanezumab is an example of this treatment strategy. This immune drug that removes Aβ from the AD brain into blood has been shown to delay cognitive decline in those in an early stage of AD [18].

In humans the relationship between amylin and Aβ in plasma is unclear. It will be important to determine whether this naturally occurring peptide derived from the pancreas has any role in regulating Aβ in the brain. If our mouse finding indicating that peripheral amylin passes through the BBB and removes Aβ from the brain is relevant to humans, we anticipate that amylin will be positively associated with Aβ in human plasma samples. Apolipoprotein E4 (ApoE4) is the major risk factor for AD with late onset [19]. The ApoE4 allele is associated with BBB damage [20], which likely adversely affects removal of Aβ from brain into blood. Using a large, homebound elderly population we aimed to examine the relationship between amylin and Aβ in blood in the context of the ApoE alleles.

## Materials and Methods

### Study Population and Recruitment

We studied a group of 1092 subjects, all of whom had measurements of plasma amylin and Aβ, as well as ApoE genotyping as part of a population-based study, the *Nutrition, Aging and Memory in the Elderly (NAME) study* [21]. Subjects included homebound elderly clients who were enrolled in one of four homecare agencies in the Boston area between 2002 and 2007. Anyone receiving homecare services was registered with one of these agencies if he/she lived in the city of Boston, had an annual income <$18,890, and needed homecare service. All homebound elders aged 60 and older at each of the four agencies were invited to participate in the study. All enrolled subjects gave written informed consent. The protocol, consent form and consent procedure were approved by the Institutional Review Boards of Tufts University New England Medical Center and Boston University School of Medicine. All the signed consent forms have been kept and locked in the research area.

Eligibility for enrollment required that the participants spoke English, were physically able to participate in the study home visits, and had sufficient vision and hearing to read and hear the content of the neuropsychological tests. Those with Mini-Mental State Examination (MMSE) ≤10 or verbal IQ<75 were not eligible to continue in the study. Of all eligible subjects, 66% enrolled in the study, and gave informed consent [22]. The subjects were screened for cognitive impairment using the Mini Mental State Examination (MMSE) [23].

### Measurements

#### Plasma Amylin and Aβ

Fasting blood draws were conducted. Blood samples were centrifuged immediately following blood draw to isolate plasma. We used ELISA assay to measure amylin concentration in plasma according to the manufacture's instructions (LINCO Research, St. Charles, Missouri). All samples were assayed in duplicate and averaged to give final values.

To measure Aβ a sandwich Aβ ELISA was used, as described previously [24]. Briefly, plates were coated with 2G3 (anti-Aβ40) and 21F12 (anti-Aβ42) antibodies overnight at 4°C. Samples were then loaded and incubated overnight at 4°C followed by incubation with a biotinylated monoclonal anti-N terminus Aβ antibody (3D6B) for 2 hrs. Finally, streptavidin-conjugated alkaline phosphatase (Promega, USA) was added and incubated, and the signal was amplified by adding alkaline phosphatase fluorescent substrate (Promega, USA), which was then measured.

#### ApoE genotyping

A 244 bp fragment of the apoE gene including the two polymorphic sites was amplified by PCR using a robotic Thermal Cycler (ABI 877, Perkin-Elmer/Applied Biosystems), using oligonucleotide primers F4 (5′-ACAGAATTCGCCCCGGCCTGGTACAC-3′) and F6 (5′-TAAGCTTGGCACGGCTGTCCAAGGA-3′). The PCR products were digested with 5 units of Hha I and the fragments separated by electrophoresis on 8% polyacrylamide non-denaturing gel. The specific allelic fragments were: E2; E3; and E4. ApoE4 was defined by E4/4, E3/4 or E2/4 [25].

#### Other blood tests

Serum lipid profiles, including cholesterol, LDL and HDL, and serum creatinine, were measured by the clinical laboratory according to the standard protocols at Jean Mayer USDA Human Nutrition Research Center on Aging (HNRCA), Tufts University.

### Other Clinical Evaluation

Weight and height were measured twice using standardized instruments for weight and height, and the average of two measurements was used to calculate BMI (kg/m^2^). Diabetes was defined by the use of anti-diabetic medication or fasting glucose greater than 126 mg/dl, parameters widely used in population-based studies [26]. Subjects were asked to show all the medications they were taking, and research assistants documented the medication names according to the labels.

### Statistical Analysis

Statistical analysis was performed using SAS (version 9.1). Normally distributed variables, such as age, were presented as mean ± SD and compared using t-tests for the ApoE4 subgroups or using ANOVA test across the quartiles. Variables with skewed distributions (plasma amylin, Aβ1-42, Aβ1-40, and Aβ1-40/Aβ1-42 ratio) were presented as median (25^th^, 75^th^ percentiles) and compared using Wilcoxon rank sum test for ApoE4 subgroups or using Kruskal-Wallis test across quartiles [24]. The Chi-Square test was used to compare proportions for binary endpoints. Amylin (LogAmylin), Aβ1-42 (Log Aβ1-42), and Aβ1-40 (Log Aβ1-40) were transformed to log_10_ for multivariate regression due to skewed distributions. Univariate and multivariate linear regression were used to examine associations between Log Amylin and Log Aβ1-42 or Log Aβ1-40 while adjusting for age, ApoE4, depression, creatinine and other confounders. For all analyses, the two-sided significance level of 0.05 was used.

## Results

### Study Population

From the completed NAME study, 1092 subjects with ApoE genotyping and measurements of plasma amylin and Aβ were used for the study analysis (Table 1); 24% of them carried at least one ApoE4 allele. The average age (mean ± SD) of this study sample was 75.0±8.0 years old, and 76% were female. The population was multi-ethnic, with 61% Caucasian, 35% African American and 4% other ethnicities. Most subjects (67%) had high school education or above. The average BMI was 31.6±8.6, and 37% had a history of diabetes (Table 1). The distributions of all amylin, Aβ1-42 and Aβ1-40 in plasma were skewed (Table 1). For plasma amylin (pM/L): median  = 22.3, Q1 = 11.8, Q3 = 40.0; for Aβ1-42 (pg/ml): median  = 17.4, Q1 = 11.8, Q3 = 22.6, and for Aβ1-40 (pg/ml): median  = 133.4, Q1 = 99.5, Q3 = 172.7.

**Table 1:** General information and univariate correlations of amylin and Aβ stratified by ApoE4 status.

	All subjects	ApoE4 Non-carriers	ApoE4 Carriers	
	n = 1092	n = 834	n = 258	
**General Information**				
**Age, year, mean ± SD**	75.0±8.0	75.2±8.5	74.3±8.4	
**Female, n/total (%)**	831/1092 (76%)	628/834 (75%)	203/258 (79%)	
**African Americans, n/total (%)**	383/1087 (35%)	258/829 (31%)	125/258 (48%)***	
**High School Graduate and above, n/total (%)**	726/1086 (67%)	555/832 (67%)	171/254 (67%)	
**History of stroke, n/total (%)**	216/1062 (20%)	170/813 (21%)	46/249 (18%)	
**BMI, mean ± SD**	31.6±8.6	31.7±8.9	31.3±8.0	
**Diabetes, n/total (%)**	387/1059 (37%)	293/810 (36%)	94/249 (38%)	
**Creatinin, mean ± SD**	1.1±1.0	1.0±0.8	1.2±1.5	
**Cholesterol, mg/dL, Mean ± SD**	184.9±43.2	184.4±43.5	187.0±42.3	
**LDL, mg/dL, Mean ± SD**	107.3±35.9	106.4±36.1	110.4±35.4	
**HDL, mg/dL, Mean ± SD**	49.6±14.8	49.4±15.0	50.4±14.1	
**Amylin and Aβ in Plasma**				
**Amylin, pM/L, Median (Q1, Q3)**	22.3 (11.8, 40.0)	20.4 (11.8, 38.7)	22.5 (11.5, 41.2)	
**Aβ1- 42, pg/ml, Median (Q1, Q3)**	17.4 (11.8, 22.6)	18.0 (12.2, 27.7)	17.1 (11.4, 23.5)*	
**Aβ1- 40, pg/ml, Median (Q1, Q3)**	133.4 (99.5, 172.7)	134.0 (99.7, 174.1)	135.2 (100.3, 183.8)	
**Aβ1- 40/Aβ1- 42 ratio, Median (Q1, Q3)**	7.6 (4.9, 11.0)	7.4 (4.7, 10.7)	8.8 (5.3, 11.9)**	
**Univariate Spearman Correlations**				
**Amylin with Aβ1- 42**	r = +0.20, p<0.0001	r = +0.21, p<0.0001	r = +0.22, p = 0.0003	
**Amylin with Aβ1- 40**	r = +0.12, p<0.0001	r = +0.15, p<0.0001	r = +0.04, p = 0.57	

Mean ± SD or n/total (%) and the comparisons between ApoE4 non-carriers and ApoE4 carriers are presented. P values for the statistical significance are shown.

*p = 0.03;

**p = 0.007;

***p<0.0001.

Univariate Spearman Correlations on the relationships between amylin and Aβ are shown for the whole sample and the ApoE4 subgroups.

BMI  =  Body Mass Index; LDL  =  low density lipoprotein; HDL  =  high density lipoprotein.

We divided subjects into ApoE4 non-carriers (n = 834) and ApoE4 carriers (n = 258) (Table 1). There were no differences in demographic variables between the two ApoE subgroups with the exception that African Americans were more likely to be ApoE4 carriers than ApoE4 non-carriers (48% vs. 31%, p = 0.03). There were no differences in lipid profiles between the two ApoE4 subgroups. Compared to the ApoE4 non-carriers, ApoE4 carriers had a slightly lower concentration of Aβ1-42 in plasma (p = 0.03) and a higher Aβ1-40/Aβ1-42 ratio (p = 0.007). There were no differences in the concentrations of amylin and Aβ1-40 in the ApoE4 subgroups.

### A Positive Relationship between Plasma Amylin and Aβ

Using Spearman rank correlation analysis, the study sample showed that amylin was positively and moderately associated with Aβ1-42 (r = +0.20, p<0.0001) and Aβ1-40 (r = +0.12, p<0.0001) in plasma (Table 1). Subjects were divided into quartiles based on the concentration of plasma amylin (Table 2). In plasma with increasing quartile of amylin the concentrations of Aβ1-42 (p<0.0001) increased in a linear pattern; the concentrations of Aβ1-40 (p<0.0001) also increased, but in a non-linear pattern with a U shape; and the ratios of Aβ1-40/Aβ1-42 (p<0.0001) decreased in a linear pattern.

**Table 2:** Comparisons of Aβ1-42, Aβ1-40 and Aβ1-40/Aβ1-42 ratio across the amylin quartiles.

Amylin Quartiles	Quartile 1	Quartile 2	Quartile 3	Quartile 4	P values
**Aβ1- 42, Median**	16.0	16.3	17.1	21.4	
**(Q1, Q3)**	(11.4, 22.3)	(11.4, 24.2)	(12.2, 26.1)	(13.7, 39.7)	<0.0001
**Aβ1- 40, Median**	134.4	126.8	127.0	150.2	
**(Q1, Q3)**	(101.9, 176.1)	(91.0, 166.8)	(91.4, 160.5)	(117.2, 160.5)	<0.0001
**Aβ1-40/Aβ1- 42, Median**	9.0	7.8	7.1	6.9	
**(Q1, Q3)**	(6.03, 12.6)	(5.1, 11.2)	(4.6, 10.3)	(4.3, 10.0)	<0.0001

Median (Q1, Q3) is used to describe the distributions of plasma Aβ1-42, Aβ1-40 and the Aβ1-40/Aβ1-42 ratio. Using Kruskal-Wallis test, the comparisons are shown across the amylin quartiles. P values for the statistical significance are shown.

While there was no difference in age across the four amylin quartiles, average BMI (p<0.0001), kidney function as assessed by creatinine levels (p<0.0001), and the rate of diabetes (p<0.05) all increased with increasing quartile of amylin (Table 3). Cholesterol and LDL levels had a positive relationship with increasing 1^st^ to 3^rd^ quartile of amylin, but their levels were lower in the 4^th^ quartile of amylin, indicating a nonlinear relationship. HDL concentration was inversely associated with increasing quartile of amylin in plasma (P<0.0001).

**Table 3:** Comparisons of age, metabolic diseases and biomarkers across the amylin quartiles.

Amylin Quartiles	Quartile 1	Quartile 2	Quartile 3	Quartile 4
**Age, year, Mean ± SD**	75.5±8.7	75.0±8.4	75.1±8.5	74.2±8.5
**History of stroke, n/total (%)**	54/265 (20%)	61/272 (22%)	53/270 (20%)	50/268 (19%)
**BMI, Mean ± SD** **	30.0±8.4	31.4±9.0	32.0±7.6	32.9±9.1
**Diabetes, n/total (%)** *	97/265 (37%)	87/269 (32%)	92/268 (34%)	117/265 (44%)
**Creatinine, mg/dL, Mean ± SD** ***	0.90±0.68	0.93±0.92	1.05±0.74	1.39±1.53
**Cholesterol, mg/dL, Mean ± SD** **	183.8±46.3	183.8±41.3	192.1±43.9	179.0±40.2
**LDL, mg/dL, Mean ± SD** **	106.7±38.1	108.1±35.1	112.4±35.6	100.9±33.5
**HDL, mg/dL, Mean ± SD** ***	53.6±16.6	49.3±13.7	49.2±14.7	46.6±13.4

Mean ± SD with ANOVA test or n/total with Chi-Square test is used to describe the distributions and comparisons of age, diseases and the biomarkers across the amylin quartiles.

*p≤.05.

**p≤.001,

***p≤.0001 for the statistical significance are shown.

BMI  =  Body Mass Index; LDL  =  low density lipoprotein; HDL  =  high density lipoprotein.

Because of their skewed distributions, amylin, Aβ1-42 and Aβ1-40 were transformed to log10 for multivariate linear regression. Using multivariate regression (Table 4), log_10_ of amylin remained positively associated with both log_10_ of plasma Aβ1-42 (β = +0.166, SE = 0.024, P<0.0001) and log_10_ of plasma Aβ1-40 (β = +0.053, SE = 0.016, P = 0.001) respectively after adjusting for age, gender, ethnicity, BMI and ApoE4 (Model I). After adding kidney function as assessed by concentration of creatinine, log_10_ of amylin was still associated with either log_10_ of plasma Aβ1-42 (β = +0.150, SE = 0.024, P<0.0001) or log_10_ of plasma Aβ1-40 (β = +0.031, SE = 0.016, P = 0.05) (Model II), but the relationships were attenuated. Finally, the relationship between log_10_ of amylin and either log_10_ of plasma Aβ1-42 (β = +0.146, SE = 0.025, P<0.0001) or log_10_ of plasma Aβ1-40 (β = +0.034, SE = 0.016, P = 0.04) as an outcome persisted after adjusting for age, gender, ethnicity, ApoE4, BMI, diabetes, stroke, creatinine and lipid profile including cholesterol, LDL and HDL (Model III). Adding education and MMSE to the model did not change the relationships between amylin and Aβ (data not shown). The concentration of creatinine was positively associated with both log_10_ of plasma Aβ1-40 and log_10_ of plasma Aβ1-42. ApoE4 was negatively associated with plasma Aβ1-42, but not with plasma Aβ1-40 in this model.

**Table 4:** Effects of amylin on Aβ42 or Aβ42 in plasma in multivariate regression analyses.

Whole sample	Log Aβ1-42		Log Aβ1-40	
	Estimate β (SE)	P value	Estimate β (SE)	P value
**Model I: Log Amylin**	+0.166 (0.024)	<0.0001	+0.053 (0.016)	0.001
**Model II: Log Amylin**	+0.150 (0.024)	<0.0001	+0.031 (0.016)	0.05
**Model III: Log Amylin**	+0.149 (0.025)	<0.0001	+0.034 (0.016)	0.04

Model I: adjusting for age, gender, ethnicity, BMI and ApoE4; n = 1000 for Aβ1-42; n = 1001 for Aβ1-40.

Model II: Model I plus creatinine; n = 983 for Aβ1-42; n = 984 for Aβ1-40.

Model III: Model II plus diabetes, stroke, cholesterol, LDL and HDL; n = 951 for Aβ1-42; n = 952 for Aβ1-40.

### The Relationship between Amylin and Aβ in the Context of ApoE Allele

**Figure 1:**
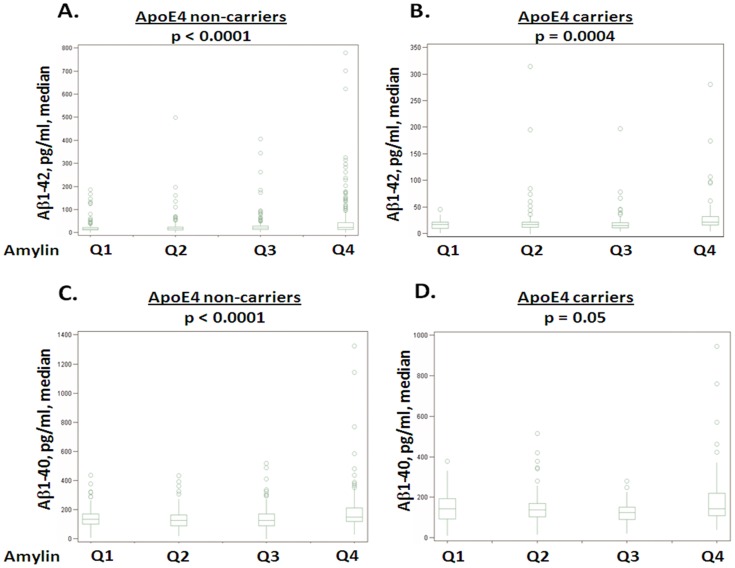
Characterization of Aβ with increasing amylin quartiles. Subjects are divided into four quartiles based on the concentration of amylin in plasma. Box plots were used to illustrate the median concentration with 25% (Q1) and 75% (Q3) range of plasma Aβ. Plasma Aβ1-42 (A and B) and Aβ1-40 (C and D) in the absence (A and C) and presence (B and D) of ApoE4 allele in quartile 1 (Q1), quartile 2 (Q2), quartile 3 (Q3) and quartile 4 (Q4) of plasma amylin are shown.

Subjects were divided into ApoE4 non-carriers and carriers (Table 1). In the absence of ApoE4, the relationships between amylin and Aβ1-42 (β = +0.158, SE = 0.031, P<0.0001) or Aβ1-40 (β = +0.044, SE = 0.020, P = 0.03) in plasma still remained after adjusting for age, gender, ethnicity, BMI, diabetes, stroke, creatinine and the lipid profile (Tables 1 and 5). With increasing quartile of amylin, the concentrations of Aβ1-42 (p<0.0001) (Figure 1A) and Aβ1-40 (p<0.0001) (Figure 1C) increased in plasma in ApoE4 non-carriers. Since increased amylin was more associated with Aβ1-42 than with Aβ1-40, the ratio of Aβ1-40/Aβ1-42 was decreased with increasing quartile of amylin in the absence (median: Q1 = 8.7; Q2 = 7.7; Q3 = 7.0 and Q4 = 6.6, p = 0.0001).

**Table 5:** Effects of amylin on Aβ42 or Aβ42 in plasma in the absence and the presence of ApoE4 allele.

ApoE4 non-carriers	Log Aβ1-42 (n = 746)		Log Aβ1-40 (n = 746)	
	Estimate β (SE)	P value	Estimate β (SE)	P value
**Log Amylin**	+0.158 (0.031)	<0.0001	+0.044 (0.020)	0.03

ApoE4 carriers	Log Aβ1-42 (n = 229)		Log Aβ1-40 (n = 230)	
	Estimate β (SE)	P value	Estimate β (SE)	P value
**Log Amylin**	+0.112 (0.042)	0.008	−0.001 (0.031)	0.98

Adjusted for age, gender, ethnicity, BMI, diabetes, stroke, creatinine, cholesterol, LDL and HDL for the ApoE subgroups.

In contrast to ApoE4 non-carriers, ApoE4 carriers had a different pattern of the relationship between amylin and Aβ. In the presence of ApoE4, while the relationship between amylin and Aβ1-42 (β = +0.112, SE = 0.042, P = 0.008) remained but attenuated, the relationship between amylin and Aβ1-40 disappeared after adjusting for the confounders (Tables 1 and 5). In ApoE4 carriers, with increasing quartile of amylin, the concentrations of Aβ1-42 increased in plasma (p = 0.0004) (Figure 1B), but the relationship between amylin quartiles and Aβ1-40 in plasma was weak and presented with a U shape (Figure 1D). The ratio of Aβ1-40/Aβ1-42 was decreased with increasing quartile of amylin in the presence of ApoE4 allele (median: Q1 = 10.1; Q2 = 9.0; Q3 = 8.5 and Q4 = 7.3, p = 0.006). ApoE4 carriers had a higher level of Aβ1-40/Aβ1-42 ratio than ApoE4 non-carriers in amylin quartiles 1 and 3 with statistical significance (p<0.05).

## Discussion

Our recent study using AD mouse models demonstrated that i.p. injection of synthetic amylin or its analog, pramlintide, enhanced the removal of Aβ from the brain into blood (submitted and under review). In light of this effect, we hypothesized that endogenous amylin in blood would enhance the removal of Aβ from the brain, especially in elderly with amyloid pathology in the brain. This would lead to a positive relationship between these two peptides in blood. Our current human study did indeed show a positive association between naturally occurring amylin and Aβ in plasma, likely due to a mechanism similar to that seen in the mouse model. Thus it is possible that endogenous and synthetic amylin have similar effects on Aβ in the brain.

It is intriguing that amylin (Tables 2 and 3) and Aβ, especially Aβ1-42, were positively associated in plasma, suggesting that naturally occurring amylin may also enhance removal of Aβ from the brain. In general, a positive association between two peptides in a compartment in the body can occur by three mechanisms, e.g. 1) co-production/co-secretion, 2) competitive degradation/clearance, or 3) one peptide moving another to the same location. It is logical to anticipate that if two molecules do not encounter each other in the same tissue regions, they generally will not compete for the same protease degradation or bind the same receptor to be cleared or influence each other, and thus will not have a positive association. Although amylin is a peripheral peptide produced and secreted by the pancreas and Aβ occurs primarily in the brain, especially the AD brain [16], amylin does readily cross the BBB [7], [8] and thus amylin and Aβ may therefore encounter each other in the brain. Another pancreatic peptide, insulin, is much less likely than amylin to be transported into the brain via the BBB [7], [8]. It is not surprising that in the same plasma samples we did not find any association between insulin and Aβ (data not shown). Note that when using cell cultures, insulin and Aβ encounter each other in the cell media and a significantly positive relationship between insulin and Aβ is observed [3]–[5].

Amylin levels were inversely associated with the Aβ1-40/Aβ1-42 ratio in plasma (Table 2 and Figure 1). Two large, prospective population studies have shown that a high plasma Aβ40/Aβ42 ratio, determined by both low Aβ42 and high Aβ40, increases the risk of AD [27], [28]. While Aβ42 is a major component of AD pathology in the brain [29], Aβ40 is a component of cerebral amyloid angiopathy (CAA) [30]. High levels of plasma Aβ40 are associated with cerebral microvascular pathology, white matter hyperintensities (WHI) and lacunar infarcts [31], [32]. The plasma Aβ42 decline seen in the pre-clinical stage of AD [33], [34], indicating the formation of AD pathology. Thus a high Aβ40/Aβ42 ratio in plasma may be a biomarker of cerebral microvascular pathology, which is associated with high plasma Aβ40, co-existing AD pathology, which is associated with low plasma Aβ42. Since plasma amylin levels were found to be inversely associated with Aβ40/Aβ42 ratio, it is possible that higher plasma amylin is a protecting factor for the development of AD.

ApoE4 is a major risk factor for late-onset AD as well as for cerebrovascular disease [35]. The positive association between amylin and Aβ1-40in blood disappeared in the presence of ApoE4 (Table 5). Although the effect of ApoE4 on the relationship between amylin and Aβ is unknown, we hypothesized that ApoE4 may attenuate amylin's activity in removing Aβ, especially Aβ40, out of the brain via the BBB. Aβ40 is the primary peptide that is deposited in the cerebrovasculature of the AD brain under the influence of the ApoE4 allele [36]. BBB dysfunction, decreased cerebral blood flow, and impaired vascular clearance of Aβ from brain are all thought to contribute to AD pathogenesis [15]. Amylin has been shown to have a vasorelaxant effect [37] that may result in enhanced removal of Aβ from the brain. A recent study found an accumulation of amylin amyloid in the cerebrovasculature of the AD brain [6]; the resulting microvascular dysfunction may interfere with amylin's ability to relax cerebrovasculature. Since some ApoE4 carriers do not develop AD even at a great age [38], other factors, such as amylin, may interact with ApoE4 to influence AD development.

High plasma levels of amylin were associated with obesity and type 2 diabetes, as well as with other biomarkers of metabolic syndrome and cerebrovascular disease including low HDL levels, high creatinine levels, and non-linear increased levels of cholesterol and LDL (Table 2). These data suggest a relationship between amylin resistance, obesity, and type 2 diabetes, which is consistent with findings in other studies [39] [40] [41], [42]. Amylin was independently associated with Aβ even after adjusting for these biomarkers of metabolic syndrome (Table 3). Since amylin is cleared by the kidney [43], the relationship between plasma amylin and Aβ, especially Aβ1-40, was influenced by adding creatinine to the models.

Amylin's major role in the brain is to reduce food intake thereby controlling body weight and regulating glucose metabolism [44]. Administering exogenous amylin, either peripherally or intracerebroventricularly, results in reduced appetite and food intake [8]. Pramlintide, an amylin analog differing by three amino acids, is an effective and well-tolerated drug in clinical use for the treatment of diabetes [45] [46]. Given the effectiveness of the amylin class of peptides in reducing amyloid pathology in the brain in the preclinical study and the relationship between amylin and Aβ in the context of ApoE allele seen in this human study, pramlintide may have potential as a treatment in AD. A clinical trial of pramlintide in AD, an off-label use, may be warranted. Limitations of our study are its cross-sectional design and lack of brain imaging. Longitudinal studies are needed to confirm the causal relationship between high levels of plasma amylin and decreased Aβdeposition in the brain. There were no diagnoses of AD and mild cognitive impairment (MCI) for this population based study. Future studies are necessary to examine the concentrations of amylin and its relationship to Aβ in specific diagnostic groups.
